# RETRACTION: Reciprocal Effects of Oxidative Stress on Heme Oxygenase Expression and Activity Contributes to Reno‐Vascular Abnormalities in EC‐SOD Knockout Mice

**DOI:** 10.1155/ijhy/9783742

**Published:** 2026-03-25

**Authors:** International Journal of Hypertension

RETRACTION: T. Kawakami, N. Puri, K. Sodhi, et al., “Reciprocal Effects of Oxidative Stress on Heme Oxygenase Expression and Activity Contributes to Reno‐Vascular Abnormalities in EC‐SOD Knockout Mice,” *International Journal of Hypertension*, 2012, 740203, https://doi.org/10.1155/2012/740203.

The above article, published online on 12 January 2012 in Wiley Online Library (https://wileyonlinelibrary.com), has been retracted by agreement between the journal Editor‐in‐Chief, Franco Veglio; and John Wiley & Sons Ltd. The retraction has been agreed due to concerns identified in the Figures, both directly and on PubPeer [1]. On further investigation, the below issues have identified:

• The Actin bands shown in Figure 1b and 2j, and the AKT bands shown in Figure 4c, appear to be identical to each other.
• The Actin bands shown in Figure 1b and 2j, and the AKT bands shown in Figure 4c, appear to be identical to the Actin bands shown in Figure 2a of [2].
• The HO‐1 bands shown in Figure 1b appear to be identical to the XO bands shown in Figure 2a of [2].
• The HO‐2 bands shown in Figure 1b appear to be identical to the HO‐2 bands shown in Figure 2e of [3].

The authors were contacted regarding these concerns, and raw data plus an explanation were requested, but no response was received. The apparent duplication of bands in figures within the present article bring into question the validity of the data. Regarding the apparent duplication of bands across different articles: whilst references 2 and 3 were published after the present article, no explanation or response was received by the authors for any of the figure concerns. As such, the Editorial Board has concluded that the results and conclusions can no longer be considered reliable. The article is therefore retracted.

The authors did not respond to the retraction.
